# Mechanisms of sleep disturbances in long-term cancer survivors: a childhood cancer survivor study report

**DOI:** 10.1093/jncics/pkae010

**Published:** 2024-02-15

**Authors:** Lauren C Daniel, Huiqi Wang, Tara M Brinkman, Kathy Ruble, Eric S Zhou, Oxana Palesh, Robyn Stremler, Rebecca Howell, Daniel A Mulrooney, Valerie M Crabtree, Sogol Mostoufi-Moab, Kevin Oeffinger, Joseph Neglia, Yutaka Yasui, Gregory T Armstrong, Kevin Krull

**Affiliations:** Department of Psychology, Rutgers University Camden, Camden, NJ, USA; Department of Epidemiology and Cancer Control, St. Jude Children’s Research Hospital, Memphis, TN, USA; Department of Epidemiology and Cancer Control, St. Jude Children’s Research Hospital, Memphis, TN, USA; Department of Psychology and Biobehavioral Sciences, St. Jude Children’s Research Hospital, Memphis, TN, USA; Department of Oncology, Johns Hopkins University, Baltimore, MD, USA; Division of Sleep Medicine and Department of Psychiatry, Harvard Medical School, Boston, MA, USA; Department of Pediatrics, Dana Farber Cancer Institute, Boston, MA, USA; Department of Psychiatry, Virginia Commonwealth University Massey Cancer Center, Richmond, VA, USA; Lawrence Bloomberg Faculty of Nursing, University of Toronto, Toronto, ON, Canada; Department of Radiation Physics, MD Anderson, Houston, TX, USA; Department of Epidemiology and Cancer Control, St. Jude Children’s Research Hospital, Memphis, TN, USA; Department of Oncology, St. Jude Children’s Research Hospital, Memphis, TN, USA; Department of Psychology and Biobehavioral Sciences, St. Jude Children’s Research Hospital, Memphis, TN, USA; Division of Oncology, Children’s Hospital of Philadelphia, Philadelphia, PA, USA; Department of Pediatrics, Perelman School of Medicine of the University of Pennsylvania, Philadelphia, PA, USA; Department of Medicine, Duke University and Duke Cancer Institute, Durham, NC, USA; Department of Pediatrics, University of Minnesota Medical School, Minneapolis, MN, USA; Department of Epidemiology and Cancer Control, St. Jude Children’s Research Hospital, Memphis, TN, USA; Department of Epidemiology and Cancer Control, St. Jude Children’s Research Hospital, Memphis, TN, USA; Department of Oncology, St. Jude Children’s Research Hospital, Memphis, TN, USA; Department of Epidemiology and Cancer Control, St. Jude Children’s Research Hospital, Memphis, TN, USA; Department of Psychology and Biobehavioral Sciences, St. Jude Children’s Research Hospital, Memphis, TN, USA

## Abstract

**Background:**

Sleep problems following childhood cancer treatment may persist into adulthood, exacerbating cancer-related late effects and putting survivors at risk for poor physical and psychosocial functioning. This study examines sleep in long-term survivors and their siblings to identify risk factors and disease correlates.

**Methods:**

Childhood cancer survivors (≥5 years from diagnosis; n = 12 340; 51.5% female; mean [SD] age = 39.4 [9.6] years) and siblings (n = 2395; 57.1% female; age = 44.6 [10.5] years) participating in the Childhood Cancer Survivor Study completed the Pittsburgh Sleep Quality Index (PSQI). Multivariable Poisson-error generalized estimating equation compared prevalence of binary sleep outcomes between survivors and siblings and evaluated cancer history and chronic health conditions (CHC) for associations with sleep outcomes, adjusting for age (at diagnosis and current), sex, race/ethnicity, and body mass index.

**Results:**

Survivors were more likely to report clinically elevated composite PSQI scores (>5; 45.1% vs 40.0%, adjusted prevalence ratio [PR] = 1.20, 95% CI = 1.13 to 1.27), symptoms of insomnia (38.8% vs 32.0%, PR = 1.26, 95% CI = 1.18 to 1.35), snoring (18.0% vs 17.4%, PR = 1.11, 95% CI = 1.01 to 1.23), and sleep medication use (13.2% vs 11.5%, PR = 1.28, 95% CI = 1.12 to 1.45) compared with siblings. Within cancer survivors, PSQI scores were similar across diagnoses. Anthracycline exposure (PR = 1.13, 95% CI = 1.03 to 1.25), abdominal radiation (PR = 1.16, 95% CI = 1.04 to 1.29), and increasing CHC burden were associated with elevated PSQI scores (PRs = 1.21-1.48).

**Conclusions:**

Among survivors, sleep problems were more closely related to CHC than diagnosis or treatment history, although longitudinal research is needed to determine the direction of this association. Frequent sleep-promoting medication use suggests interest in managing sleep problems; behavioral sleep intervention is advised for long-term management.

More than 85% of children diagnosed with cancer today will become 5-year survivors (1). However, these survivors are well established to be at increased risk for severe and life-threating chronic health conditions (CHC) and early mortality (2,3). Sleep disturbances are common across the continuum of cancer survivorship (4,5), posing a threat to health and quality of life for long-term survivors. Sleep disturbances are linked to impairments in both mental and physical health (6,7) as well as earlier mortality (8) in the general population and in childhood cancer survivors (9-12).

Although sleep disturbances resolve for some survivors following the completion of therapy, obstructive sleep apnea or insomnia persists in approximately 5%-25%, respectively, of long-term childhood cancer survivors, rates that are higher than among siblings (13). Our prior work with the Childhood Cancer Survivor Study (CCSS) suggests survivors exhibit greater risk for insomnia, daytime sleepiness, snoring, and fatigue relative to siblings (13,14). Survivors who report poor sleep were also at risk for increased or persistent emotional distress and are more likely to develop migraines over time (13). However, these results (13,14) are based on a subset of cancers diagnosed from 1970 to 1986 and do not reflect those treated with more recent therapeutic protocols.

Several possible underlying mechanisms leading to sleep disturbances after cancer treatment are tested in the current study of survivors diagnosed from 1970 to 2000: (1) cancer history and its hypothesized influence on mental health elevates the risk of developing or perpetuating insomnia (15), tested by comparing insomnia symptoms between survivors and siblings; (2) hypothalamic damage from treatment or tumor location may alter the circadian regulation of sleep (16,17), tested by comparing risk of delayed bedtimes in those with and without central nervous system (CNS) related diagnoses and therapies; (3) respiratory distress because of excess weight (18) or treatment-related changes to the upper airway (19) and/or pulmonary functioning (20) (ie, head/neck or thoracic radiation) may increase risk of sleep disordered breathing, tested by examining the contribution of body mass index (BMI) to sleep outcomes, the frequency of snoring, and the contribution of pulmonary-directed therapies on snoring and sleep quality; or (4) CHC burden and cancer-related late effects (21,22), such as pain, that disrupt sleep quality. These mechanisms likely interact to exacerbate sleep disturbances for some survivors.

Understanding the continued risk for sleep disorders in childhood cancer survivors is important for the development of interventions to improve health and functional outcomes. We hypothesize that survivors will report poorer sleep quality, more symptoms of insomnia, greater symptoms of sleep-disordered breathing, more delayed sleep phase, and more frequent use of sleep medication than siblings.

## Methods

### Study population

The CCSS is a retrospective cohort with longitudinal follow-up of children diagnosed with pediatric cancer (including leukemia, CNS tumors, lymphoma, Wilms tumor, neuroblastoma, and soft tissue or bone sarcoma) before age 21 and have been off treatment for at least 5 years. Institutional review boards at the 31 member institutions approved the protocol (IRB Protocol #CR00007578), and participants completed written informed consent for data collection and medical record abstraction. A random sample of one-third of closest-age siblings was also recruited. Participants with proxy-completed measures were excluded (Consort Flow Diagrams, Supplementary Figures 1 and 2, available online).

### Measures

#### Treatment exposures

Cancer diagnosis and treatment history was systematically abstracted from medical records. Radiation dosimetry was quantified as maximum target dose (maxTD) to 4 body regions (brain, chest, neck, and abdomen). The maxTD was taken as the sum of prescribed radiation dose (23) from overlapping fields in each region, separated into none, moderate (<20 Gy cranial or <30 Gy chest, neck, abdominal), or high (≥20 Gy, or ≥30 Gy chest, neck, abdominal) doses based on Children’s Oncology Group Long-Term Follow-up Guidelines (24). For individuals who received radiation therapy to more than one region, the highest maxTD values were used in analyses.

#### Pittsburgh Sleep Quality Index (PSQI)

Participants completed the PSQI to describe sleep habits including sleep medication use over the past month on a 4-point scale, with higher scores indicating worse sleep and total scores of greater than 5 indicating clinically significant poor sleep quality (25). Additional PSQI items were dichotomized to indicate clinically significant cut points: sleep onset latency was dichotomized at 30 minutes 3+ days per week, sleep efficiency (percentage of time in bed spent asleep) was dichotomized at 85%, and regular night/early morning awakenings (>3+ days per week), consistent with the diagnostic criteria for insomnia (26). Typical bedtimes later than 1:00 am were used to indicate delayed sleep phase. Self-report of snoring 3+ nights per week is suggestive of obstructive sleep apnea (27). Sleep disturbance from pain 3+ nights was also included.

#### Chronic health conditions 

Survivors completed surveys about multiple organ system–based CHC, which were graded using the Common Terminology Criteria for Adverse Events (v4.03) as mild (grade 1), moderate (grade 2), severe (grade 3), or life-threatening/disabling (grade 4) (28). The maximum grade for each participant prior to the PSQI survey was used to classify chronic condition severity as none/mild (0 and grade 1), moderate (grade 2), and severe or life-threatening (grade 3 or 4). A composite burden score reflecting number and severity of CHC was computed, categorized as none/low (no conditions or grade 1 only), medium (≥1 grade 2 and/or 1 grade 3), high (≥2 grade 3 or 1 grade 4 and 1 grade 3 condition), and very high (≥2 grade 4 or ≥2 grade 3 and 1 grade 4 condition) (29,30).

### Statistical analysis

Demographic variables (age, sex, race/ethnicity, BMI) were summarized in survivors and siblings separately and treatment variables in survivors only. Sleep quality (dichotomized PSQI composite scores), symptoms of insomnia (dichotomized sleep onset latency, sleep efficiency, and night awakenings), symptoms of sleep disordered breathing (dichotomized snoring), delayed sleep phase (dichotomized sleep onset/offset), and sleep medication use (dichotomized 3+ days per week vs no use) were compared between survivors and siblings. Comparisons used multivariable Poisson regression with robust sandwich variance estimates (31) to account for potential intra-family correlation, adjusting for age, sex, race/ethnicity, and BMI, which provided adjusted prevalence ratios (PRs) of sleep outcomes and 95% confidence intervals (CIs). Means of continuous variables were compared between survivors and siblings using linear models with generalized estimating equation modifications to account for potential intrafamily correlation.

Within survivors, two separate multivariable Poisson regression with robust sandwich variance estimates were used to estimate prevalence ratios of the sleep outcomes. One model included demographics and cancer diagnosis (CNS tumors were the reference group to test the hypothesis that CNS diagnoses confer additional risk for sleep) (16,17), and the other model included demographics and treatment exposures; this approach reduced confounding between diagnosis and treatments. Inverse probability weighting was applied to multivariable models to account for undersampling of acute lymphoblastic leukemia survivors in the CCSS expansion cohort (diagnosed 1987-1999).

All analyses were conducted using SAS version 9.4, and two-sided *P* values less than .05 were considered statistically significant.

## Results

### Demographic and sleep characteristics

Survivors were 39.4 years old on average (SD = 9.6), with more than 17 years since diagnosis (average = 30.9 years, SD = 7.9; Table 1). Leukemia was the most common diagnostic category (31.9%). Siblings were 57.1% female, primarily White (89.1%), and 44.6 years old on average (SD = 10.5).

**Table 1.** Demographic and treatment characteristics of survivors of childhood cancer and siblings

Characteristic	**Survivors** **(N = 12** **340)** **No. (%)**	**Siblings** **(N = 2395)** **No. (%)**
Sex		
Male	5990 (48.5)	1027 (42.9)
Female	6350 (51.5)	1368 (57.1)
Race/Ethnicity		
American Indian/Alaska Native	45 (0.4)	8 (0.3)
Asian or Pacific Islander	189 (1.5)	23 (1.0)
Black	598 (4.8)	51 (2.1)
Hispanic	881 (7.1)	82 (3.4)
White	10 439 (84.6)	2134 (89.1)
Other^a^	188 (1.5)	97 (4.1)
Age at questionnaire		
18-29	2243 (18.2)	219 (9.1)
30-39	4549 (36.9)	619 (25.8)
40-49	3644 (29.5)	774 (32.3)
50+	1904 (15.4)	783 (32.7)
Body mass index		
Normal/underweight (BMI <25)	5224 (43.1)	932 (39.4)
Overweight (BMI 25-29.9)	3770 (31.1)	761 (32.2)
Obesity (BMI ≥30)	3119 (25.7)	672 (28.4)
Age at Diagnosis		
0-4	4608 (37.3)	—
5-9	2759 (22.4)	—
10-14	2823 (22.9)	—
15+	2150 (17.4)	—
Diagnosis		
Leukemia	3935 (31.9)	—
CNS tumor	1837 (14.9)	—
Hodgkin lymphoma	1485 (12.0)	—
Non-Hodgkin lymphoma	1086 (8.8)	—
Wilms tumor	1208 (9.8)	—
Neuroblastoma	896 (7.3)	—
Soft tissue sarcoma	806 (6.5)	—
Bone cancer	1087 (8.8)	—
Chemotherapy		
No	2028 (17.1)	—
Yes	9834 (82.9)	—
Alkylating agent (cyclophosphamide equivalent dose)		
None	5444 (48.7)	—
>0 to <4000 mg/m^2^	1464 (13.1)	—
≥4000 to <8000 mg/m^2^	1579 (14.1)	—
≥8000 mg/m^2^	2699 (24.1)	—
Anthracyclines (doxorubicin equivalent dose)		
None	5715 (49.8)	—
1-249 mg/m^2^	3838 (33.4)	—
≥250 mg/m^2^	1922 (16.7)	—
Vincristine		
No	3607 (31.3)	—
Yes	7908 (68.7)	—
Vinblastine		
No	11 089 (93.6)	—
Yes	760 (6.4)	—
Corticosteroids		
No	6649 (56.2)	—
Yes	5177 (43.8)	—
Platinum agents		
No	10 594 (89.3)	—
Yes	1264 (10.7)	—
Cranial radiation		
None	8609 (74.2)	—
<20 Gy	1135 (9.8)	—
≥20 Gy	1860 (16.0)	—
Neck radiation		
None	9324 (80.3)	—
<30 Gy	1315 (11.3)	—
≥30 Gy	971 (8.4)	—
Chest radiation		
None	8970 (77.3)	—
<30 Gy	1604 (13.8)	—
≥30 Gy	1030 (8.9)	—
Abdominal radiation		
None	9195 (79.2)	—
<30 Gy	1563 (13.5)	—
≥30 Gy	851 (7.3)	—

a Participants self-identified with another racial group than options presented. BMI = body mass index; CNS = central nervous system.

Survivors reported an average nightly sleep duration of 6.9 hours (SD = 1.6) and an average PSQI total score of 5.9 (SD = 3.7). Siblings reported an average nightly sleep duration of 6.8 hours (SD = 1.3) and an average PSQI total score of 5.4 (SD = 3.4).

### Survivor and sibling comparison

**Figure 1.**
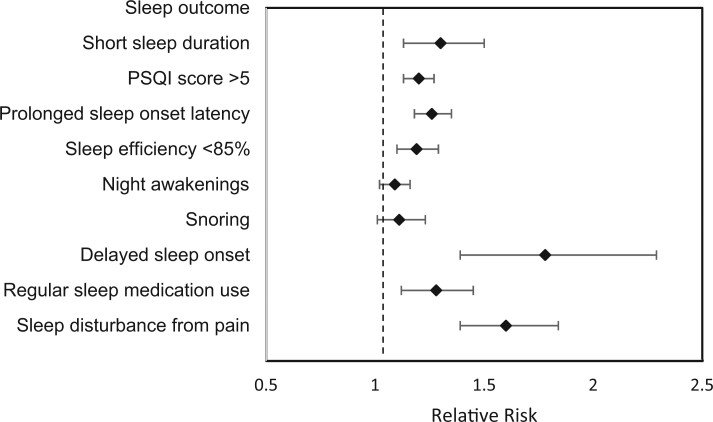
Risk of sleep concerns for survivors of childhood cancer relative to siblings. This figure shows data from modified Poisson regressions adjusted for age, sex, race, and body mass index with generalized estimating equation used to account for within-family correction. PSQI = Pittsburgh Sleep Quality Index.

Survivors were more likely to be male, be younger, and have an average range BMI (BMI < 25) and were less likely to be White than siblings (*P* < .05). After adjusting models for current age, sex, race/ethnicity, and BMI, survivors were more likely to report sleep duration of less than 6 hours (12% of survivors vs 10.6% of siblings; PR = 1.30, 95% CI = 1.13 to 1.50) and elevated PSQI total scores (45.1% of survivors vs 40.0% of siblings; PR = 1.20, 95% CI = 1.13 to 1.27). Survivors were more likely to report prolonged sleep onset latency (38.8% vs 32.0%; PR = 1.26, 95% CI = 1.18 to 1.35) and sleep efficiency less than 85% (33.3% vs 29.9%; PR = 1.19, 95% CI = 1.10 to 1.29). Frequent night/early morning awakening was similar between groups, but, after adjustment, survivors were more likely to report these awakenings (35.6% vs 36.0%; PR = 1.09, 95% CI = 1.02 to 1.16). Survivors reported more frequent snoring (18.0% vs 17.4%; PR = 1.11, 95% CI = 1.01 to 1.23). Sleep timing was also delayed, as indicated by more late bedtimes in survivors (6.2% vs 3.5%; PR = 1.78, 95% CI = 1.39 to 2.29). Survivors were more likely to report regular sleep medication use (3+ times per week; 13.2% vs 11.5%; PR = 1.28, 95% CI = 1.12 to 1.45). Survivors also reported more frequent sleep disturbance because of pain (12.5% vs 9.2%, PR = 1.60, 95% CI = 1.39 to 1.84). Sleep outcome comparisons are presented in Figure 1 and Supplementary Table 1 (available online).

### Demographic and treatment correlates of sleep

Across multivariable models (Demographic + Diagnosis, Table 2; Demographic + Treatment Exposures, Table 3), female sex was related to increased prevalence of poor sleep quality and sleep medication use and decreased risk of snoring. Overweight BMI (BMI between 25 and 29.9) increased the risk of snoring, and obese range BMI (BMI ≥ 30) was associated with increased prevalence of poor sleep quality, snoring, sleep medication use, and short sleep duration. Older age was associated with increased prevalence of snoring and decreased prevalence of later bedtime. Older individuals were more likely to report short sleep than adults aged 18-29. Survivors who were 10-14 years old at diagnosis reported increased prevalence of poor sleep quality, sleep medication use, and short sleep relative to those diagnosed at age 0-4. Those older than 15 years at diagnosis also reported higher prevalence of using sleep medications.

**Table 2.** Multivariable analysis including demographic and diagnosis as predictors of sleep outcomes

Parameter	Category	**Sleep Quality PSQI >5** ^a^ ^,^ ^b^ PR (95% CI)	**Snoring ≥3 times per week** ^a^ ^,^ ^b^ PR (95% CI)	**Bedtime after 1 am** ^a^ ^,^ ^b^ PR (95% CI)	**Any medication use** ^a^ ^,^ ^b^ PR (95% CI)	**Total sleep time <6 hours** ^a^ ^,^ ^b^ PR (95% CI)
Sex	Male	1.00 (Referent)	1.00 (Referent)	1.00 (Referent)	1.00 (Referent)	1.00 (Referent)
	Female	**1.30 (1.22 to 1.39)**	**0.67 (0.60 to 0.75)**	0.81 (0.65 to 1.01)	**1.29 (1.18 to 1.41)**	1.11 (0.97 to 1.28)
BMI	Normal/underweight	1.00 (Referent)	1.00 (Referent)	1.00 (Referent)	1.00 (Referent)	1.00 (Referent)
	Overweight	1.07 (0.99 to 1.17)	**1.67 (1.43 to 1.95)**	0.92 (0.71 to 1.19)	1.05 (0.94 to 1.16)	1.11 (0.93 to 1.32)
	Obesity	**1.30 (1.21 to 1.40)**	**2.39 (2.06 to 2.79)**	1.30 (1.00 to 1.70)	**1.22 (1.10 to 1.35)**	**1.56 (1.31 to 1.85)**
Race/Ethnicity	White	1.00 (Referent)	1.00 (Referent)	1.00 (Referent)	1.00 (Referent)	1.00 (Referent)
	American Indian/Alaska Native	**1.43 (1.12 to 1.84)**	1.50 (0.80 to 2.79)	1.81 (0.74 to 4.42)	1.35 (0.84 to 2.18)	1.64 (0.82 to 3.28)
	Asian or Pacific Islander	0.86 (0.69 to 1.08)	0.69 (0.43 to 1.09)	1.60 (0.95 to 2.67)	**0.61 (0.42 to 0.87)**	1.20 (0.74 to 1.96)
	Black	0.94 (0.78 to 1.14)	1.27 (0.94 to 1.72)	**1.92 (1.21 to 3.06)**	**0.73 (0.57 to 0.92)**	**1.54 (1.21 to 1.96)**
	Hispanic	0.95 (0.84 to 1.07)	0.89 (0.72 to 1.10)	1.29 (0.95 to 1.76)	**0.75 (0.62 to 0.90)**	**1.32 (1.05 to 1.65)**
	Other	**1.24 (1.04 to 1.46)**	1.07 (0.74 to 1.54)	**2.38 (1.53 to 3.68)**	**1.39 (1.06 to 1.82)**	1.29 (0.81 to 2.05)
Age	18-29	1.00 (Referent)	1.00 (Referent)	1.00 (Referent)	1.00 (Referent)	1.00 (Referent)
	30-39	0.97 (0.89 to 1.06)	**1.39 (1.16 to 1.68)**	**0.69 (0.53 to 0.89)**	1.02 (0.89 to 1.15)	1.18 (0.96 to 1.44)
	40-49	0.99 (0.88 to 1.10)	**1.63 (1.32 to 2.01)**	**0.61 (0.39 to 0.95)**	0.98 (0.85 to 1.14)	**1.29 (1.04 to 1.61)**
	50+	1.03 (0.91 to 1.15)	**1.67 (1.32 to 2.11)**	**0.49 (0.32 to 0.75)**	1.02 (0.87 to 1.20)	1.27 (0.94 to 1.70)
Age at diagnosis	0-4	1.00 (Referent)	1.00 (Referent)	1.00 (Referent)	1.00 (Referent)	1.00 (Referent)
	5-9	1.06 (0.96 to 1.17)	0.92 (0.78 to 1.08)	1.12 (0.82 to 1.53)	1.04 (0.91 to 1.19)	1.07 (0.88 to 1.30)
	10-14	**1.15 (1.05 to 1.26)**	1.01 (0.85 to 1.20)	1.13 (0.81 to 1.57)	**1.19 (1.04 to 1.35)**	**1.32 (1.05 to 1.65)**
	15+	1.09 (0.99 to 1.20)	0.89 (0.74 to 1.07)	0.82 (0.56 to 1.19)	**1.17 (1.02 to 1.35)**	0.89 (0.71 to 1.12)
Diagnosis	CNS tumor	1.00 (Referent)	1.00 (Referent)	1.00 (Referent)	1.00 (Referent)	1.00 (Referent)
	Leukemia	1.05 (0.97 to 1.14)	**1.22 (1.05 to 1.43)**	**1.34 (1.02 to 1.78)**	1.06 (0.94 to 1.20)	1.16 (0.95 to 1.41)
	Hodgkin disease	1.02 (0.93 to 1.12)	1.16 (0.96 to 1.40)	**1.44 (1.02 to 2.03)**	1.05 (0.91 to 1.20)	1.12 (0.89 to 1.40)
	Non-Hodgkin lymphoma	1.04 (0.94 to 1.14)	**1.23 (1.03 to 1.49)**	1.28 (0.91 to 1.80)	1.04 (0.90 to 1.21)	1.18 (0.94 to 1.47)
	Wilms tumor	0.96 (0.84 to 1.09)	**1.32 (1.04 to 1.68)**	1.05 (0.70 to 1.56)	0.92 (0.77 to 1.09)	0.89 (0.67 to 1.18)
	Neuroblastoma	0.99 (0.86 to 1.14)	1.19 (0.90 to 1.56)	1.12 (0.76 to 1.66)	1.04 (0.86 to 1.25)	0.92 (0.67 to 1.27)
	Soft tissue sarcoma	1.03 (0.90 to 1.18)	**1.31 (1.04 to 1.65)**	**1.72 (1.13 to 2.62)**	1.12 (0.93 to 1.35)	1.10 (0.83 to 1.47)
	Bone cancer	1.10 (0.99 to 1.22)	**1.24 (1.01 to 1.52)**	1.21 (0.82 to 1.80)	**1.18 (1.02 to 1.38)**	1.21 (0.94 to 1.54)

a Modified Poisson model was used to directly estimate prevalence ratio. Bolded values are significant. BMI = body mass index; CNS = central nervous system; PR = prevalence ratio; PSQI = Pittsburgh Sleep Quality Index.

b Inverse probability weighting was applied to account for the undersampling of acute lymphoblastic leukemia survivors in the design of the Childhood Cancer Survivor Study expansion cohort (diagnosis in 1987-1999).

**Table 3.** Multivariable analysis including demographic and treatment exposure predictor of sleep outcomes

Parameter	Category	**Sleep Quality PSQI >5** ^a^ ^,^ ^b^ PR (95% CI)	**Snoring ≥3 times per week** ^a^ ^,^ ^b^ PR (95% CI)	**Bedtime after 1 s** ^a^ ^,^ ^b^ PR (95% CI)	**Any medication use** ^a^ ^,^ ^b^ PR (95% CI)	**Total sleep time <6 hours** ^a^ ^,^ ^b^ PR (95% CI)
Sex	Male	1.00 (Referent)	1.00 (Referent)	1.00 (Referent)	1.00 (Referent)	1.00 (Referent)
	Female	**1.29 (1.21 to 1.38)**	**0.68 (0.61 to 0.77)**	**0.78 (0.61 to 0.99)**	**1.30 (1.18 to 1.42)**	1.10 (0.95 to 1.28)
BMI	Normal/underweight	1.00 (Referent)	1.00 (Referent)	1.00 (Referent)	1.00 (Referent)	1.00 (Referent)
	Overweight	1.08 (1.00 to 1.18)	**1.69 (1.43 to 1.99)**	0.90 (0.68 to 1.19)	1.02 (0.91 to 1.14)	1.06 (0.88 to 1.28)
	Obesity	**1.32 (1.22 to 1.43)**	**2.35 (1.99 to 2.77)**	1.25 (0.94 to 1.67)	**1.24 (1.12 to 1.38)**	**1.45 (1.20 to 1.75)**
Race/Ethnicity	White	1.00 (Referent)	1.00 (Referent)	1.00 (Referent)	1.00 (Referent)	1.00 (Referent)
	American Indian/Alaska Native	**1.52 (1.18 to 1.97)**	1.69 (0.90 to 3.17)	**1.56 (0.53 to 4.59)**	1.55 (0.95 to 2.51)	1.61 (0.75 to 3.47)
	Asian or Pacific Islander	0.91 (0.72 to 1.16)	0.66 (0.41 to 1.05)	1.64 (0.95 to 2.83)	**0.59 (0.40 to 0.89)**	1.32 (0.80 to 2.18)
	Black	0.96 (0.82 to 1.11)	**1.57 (1.17 to 2.09)**	**2.46 (1.57 to 3.87)**	0.82 (0.66 to 1.01)	**1.79 (1.42 to 2.27)**
	Hispanic	0.97 (0.86 to 1.09)	0.96 (0.77 to 1.19)	**1.43 (1.03 to 1.98)**	**0.70 (0.57 to 0.87)**	**1.39 (1.10 to 1.77)**
	Other	**1.25 (1.03 to 1.51)**	1.14 (0.77 to 1.69)	**2.17 (1.28 to 3.67)**	**1.44 (1.07 to 1.94)**	1.22 (0.73 to 2.04)
Age	18-29	1.00 (Referent)	1.00 (Referent)	1.00 (Referent)	1.00 (Referent)	1.00 (Referent)
	30-39	0.96 (0.88 to 1.04)	**1.45 (1.19 to 1.76)**	**0.73 (0.56 to 0.95)**	1.03 (0.90 to 1.18)	**1.31 (1.05 to 1.64)**
	40-49	0.91 (0.81 to 1.03)	**1.72 (1.38 to 2.16)**	**0.63 (0.41 to 0.96)**	0.96 (0.82 to 1.13)	**1.45 (1.13 to 1.85)**
	50+	0.96 (0.84 to 1.09)	**1.77 (1.37 to 2.28)**	**0.58 (0.37 to 0.92)**	1.05 (0.87 to 1.26)	**1.46 (1.05 to 2.02)**
Age at diagnosis	0-4	1.00 (Referent)	1.00 (Referent)	1.00 (Referent)	1.00 (Referent)	1.00 (Referent)
	5-9	1.04 (0.94 to 1.14)	0.92 (0.78 to 1.08)	1.09 (0.80 to 1.47)	1.03 (0.90 to 1.18)	1.09 (0.90 to 1.32)
	10-14	**1.19 (1.08 to 1.31)**	0.96 (0.82 to 1.14)	1.01 (0.72 to 1.41)	**1.20 (1.06 to 1.37)**	**1.45 (1.14 to 1.84)**
	15+	1.11 (0.99 to 1.24)	0.89 (0.73 to 1.08)	0.73 (0.50 to 1.06)	**1.18 (1.02 to 1.37)**	0.96 (0.75 to 1.23)
Chemotherapy	No	1.00 (Referent)	1.00 (Referent)	1.00 (Referent)	1.00 (Referent)	1.00 (Referent)
	Yes	1.03 (0.91 to 1.15)	1.07 (0.86 to 1.34)	0.99 (0.65 to 1.51)	1.02 (0.86 to 1.20)	1.17 (0.88 to 1.56)
Alkylating agent (cyclophosphamide equivalent dose)	None	1.00 (Referent)	1.00 (Referent)	1.00 (Referent)	1.00 (Referent)	1.00 (Referent)
	>0 to <4000 mg/m^2^	0.96 (0.86 to 1.07)	0.98 (0.80 to 1.19)	0.87 (0.62 to 1.22)	1.06 (0.90 to 1.25)	1.08 (0.85 to 1.37)
	≥4000 to <8000 mg/m^2^	1.00 (0.90 to 1.11)	1.10 (0.91 to 1.32)	1.03 (0.75 to 1.41)	1.11 (0.96 to 1.29)	0.99 (0.78 to 1.26)
	≥8000 mg/m^2^	0.96 (0.87 to 1.06)	0.97 (0.82 to 1.15)	0.96 (0.70 to 1.34)	1.12 (0.97 to 1.28)	0.86 (0.69 to 1.06)
Anthracyclines (doxorubicin equivalent dose)	None	1.00 (Referent)	1.00 (Referent)	1.00 (Referent)	1.00 (Referent)	1.00 (Referent)
	1-249 mg/m^2^	0.91 (0.83 to 1.01)	0.98 (0.83 to 1.16)	1.01 (0.75 to 1.35)	0.98 (0.85 to 1.12)	0.86 (0.70 to 1.05)
	≥250 mg/m^2^	**1.13 (1.03 to 1.25)**	1.01 (0.85 to 1.19)	1.37 (0.91 to 2.05)	1.11 (0.96 to 1.27)	0.98 (0.79 to 1.23)
Vincristine	No	1.00 (Referent)	1.00 (Referent)	1.00 (Referent)	1.00 (Referent)	1.00 (Referent)
	Yes	0.92 (0.84 to 1.01)	0.97 (0.81 to 1.14)	0.88 (0.68 to 1.15)	**0.85 (0.75 to 0.97)**	0.96 (0.79 to 1.18)
Vinblastine	No	1.00 (Referent)	1.00 (Referent)	1.00 (Referent)	1.00 (Referent)	1.00 (Referent)
	Yes	0.95 (0.84 to 1.08)	1.08 (0.86 to 1.36)	1.38 (0.87 to 2.19)	0.88 (0.74 to 1.06)	1.01 (0.74 to 1.39)
Platinum	No	1.00 (Referent)	1.00 (Referent)	1.00 (Referent)	1.00 (Referent)	1.00 (Referent)
	Yes	0.99 (0.88 to 1.11)	0.91 (0.74 to 1.11)	0.75 (0.53 to 1.07)	0.95 (0.81 to 1.11)	1.01 (0.79 to 1.29)
Corticosteroids	No	1.00 (Referent)	1.00 (Referent)	1.00 (Referent)	1.00 (Referent)	1.00 (Referent)
	Yes	1.08 (1.00 to 1.18)	1.06 (0.92 to 1.23)	1.14 (0.84 to 1.54)	1.08 (0.96 to 1.21)	1.03 (0.85 to 1.25)
Cranial radiation	None	1.00 (Referent)	1.00 (Referent)	1.00 (Referent)	1.00 (Referent)	1.00 (Referent)
	<20 Gy	1.00 (0.87 to 1.15)	0.88 (0.72 to 1.07)	1.39 (0.97 to 1.99)	0.89 (0.74 to 1.08)	0.89 (0.69 to 1.15)
	≥20 Gy	0.97 (0.87 to 1.08)	0.89 (0.72 to 1.09)	1.42 (0.98 to 2.05)	**0.85 (0.73 to 0.98)**	0.92 (0.72 to 1.19)
Neck radiation	None	1.00 (Referent)	1.00 (Referent)	1.00 (Referent)	1.00 (Referent)	1.00 (Referent)
	<30 Gy	0.97 (0.83 to 1.13)	**0.69 (0.53 to 0.90)**	1.32 (0.80 to 2.19)	1.00 (0.81 to 1.24)	0.91 (0.65 to 1.28)
	≥30 Gy	1.00 (0.71 to 1.39)	**0.63 (0.43 to 0.91)**	1.07 (0.46 to 2.46)	1.05 (0.74 to 1.50)	0.82 (0.50 to 1.34)
Chest radiation	None	1.00 (Referent)	1.00 (Referent)	1.00 (Referent)	1.00 (Referent)	1.00 (Referent)
	<30 Gy	0.97 (0.85 to 1.11)	1.05 (0.84 to 1.32)	0.70 (0.47 to 1.05)	1.06 (0.88 to 1.28)	0.87 (0.64 to 1.20)
	≥30 Gy	0.90 (0.66 to 1.23)	1.04 (0.73 to 1.50)	0.95 (0.48 to 1.89)	0.95 (0.67 to 1.36)	0.98 (0.62 to 1.55)
Abdominal radiation	None	1.00 (Referent)	1.00 (Referent)	1.00 (Referent)	1.00 (Referent)	1.00 (Referent)
	<30 Gy	**1.16 (1.04 to 1.29)**	**1.23 (1.02 to 1.49)**	**1.76 (1.26 to 2.45)**	1.10 (0.94 to 1.28)	1.16 (0.89 to 1.52)
	≥30 Gy	1.15 (0.99 to 1.33)	**1.38 (1.08 to 1.76)**	0.78 (0.46 to 1.31)	1.01 (0.84 to 1.22)	1.05 (0.76 to 1.44)

a Poisson model was used to directly estimate prevalence ratio. Bolded values are significant. BMI = body mass index; PR = prevalence ratio; PSQI = Pittsburgh Sleep Quality Index.

b Inverse probability weighting was applied to account for the under-sampling of acute lymphoblastic leukemia survivors in the design of the Childhood Cancer Survivor Study expansion cohort (diagnosis in 1987-1999).

When controlling for demographic variables, survivors of CNS tumors had a lower risk of snoring and delayed bedtimes than several other diagnostic groups, contrary to hypotheses (Table 2).

High anthracycline exposure was related to increased prevalence of poor sleep quality (≥250 mg/m^2^, PR = 1.13, 95% CI = 1.02 to 1.24). Vincristine was related to lower prevalence of sleep medication use (PR = 0.85, 95% CI = 0.75 to 0.97). High-dose cranial radiation was associated with reduced prevalence of sleep medication use (≥20 Gy, PR = 0.85, 95% CI = 0.73 to 0.98). Neck radiation was related to decreased prevalence of frequent snoring (<30 Gy PR = 0.69, 95% CI = 0.53 to 0.90; ≥30 Gy PR = 0.63, 95% CI = 0.43 to 0.91). Low-dose abdominal radiation was associated with increased prevalence of poor sleep quality (PR = 1.16, 95% CI = 1.04 to 1.29), snoring (PR = 1.23, 95% CI = 1.02 to 1.49), and delayed bedtime (PR = 1.76, 95% CI = 1.26 to 2.45). High-dose abdominal radiation was also associated with elevated snoring prevalence (≥30 Gy PR = 1.38, 95% CI = 1.08 to 1.76).

### CHC burden

Increasing CHC burden was associated with poor sleep quality and sleep medication use (Table 4). Survivors with medium disease burden were more likely to report snoring (PR = 1.16, 95% CI = 1.03 to 1.30), and those with high disease burden were more likely to report delayed bedtimes (PR = 1.43, 95% CI = 1.03 to 1.97).

**Table 4.** Associations among chronic health conditions and sleep outcomes within survivors^a^

Parameter	Category	N (%)	**Sleep Quality PSQI >5** ^b^ ^,^ ^c^ PR (95% CI)	**Snoring ≥3 times per week** ^b^ ^,^ ^c^ PR (95% CI)	**Bedtime after 1 AM** ^b^ ^,^ ^c^ PR (95% CI)	**Any medication use** ^b^ ^,^ ^c^ PR (95% CI)	**Total sleep time <6 hours** ^b^ ^,^ ^c^ PR (95% CI)
Total burden categorical score^d^	None/low	5238 (42.5)	1.00 (Referent)	1.00 (Referent)	1.00 (Referent)	1.00 (Referent)	1.00 (Referent)
	Medium	5221 (42.3)	**1.21 (1.13 to 1.30)**	**1.16 (1.03 to 1.30)**	1.23 (0.97 to 1.57)	**1.32 (1.20 to 1.46)**	1.11 (0.95 to 1.30)
	High	1259 (10.2)	**1.32 (1.21 to 1.44)**	0.98 (0.84 to 1.15)	**1.43 (1.03 to 1.97)**	**1.54 (1.36 to 1.74)**	1.16 (0.95 to 1.41)
	Very high	622 (5.0)	**1.48 (1.34 to 1.63)**	0.97 (0.77 to 1.21)	1.45 (0.95 to 2.21)	**1.79 (1.55 to 2.06)**	1.20 (0.92 to 1.58)

a Model adjusted by sex, body mass index, age, and race. Bolded values are significant. PR = prevalence ratio; PSQI = Pittsburgh Sleep Quality Index.

b Modified Poisson model was used to directly estimate prevalence ratio.

c Inverse probability weighting was applied to account for the under-sampling of acute lymphoblastic leukemia survivors in the design of the Childhood Cancer Survivor Study expansion cohort (diagnosis in 1987-1999).

d From Williams et al. 2021 (55): Category definitions were “none/low” for grade 1 conditions only; “medium” for ≥1 grade 2 and/or 1 grade 3 condition(s); “high” for ≥2 grade 3, or 1 grade 4 and 1 grade 3 conditions; and “very high” for ≥2 grade 4 or ≥2 grade 3 and 1 grade 4 condition(s).

## Discussion

Long-term childhood cancer survivors exhibit more sleep problems compared with siblings. Examining the subdomains of sleep quality, symptoms of insomnia at a level requiring clinical intervention were the most prevalent concerns identified. Although less common, survivors also reported greater risk of short sleep duration, sleepdisordered breathing symptoms, and delayed sleep timing than siblings. CHC increased the risk for poor sleep quality among survivors by 21%-48%, as well as regular sleep medication use by 32%-79%. These findings highlight the need to routinely assess sleep health in all survivors, particularly those with chronic comorbid health conditions. Although the prevalence of sleep disturbance in survivors was only 20% higher than in siblings, the impact of chronic sleep disturbance has potential to impact survivors more given their frequent health and behavioral complications.

With regard to purported mechanisms, cancer history elevated the risk of insomnia symptoms by 9%-26%. Although mental health was not directly measured at the time the PSQI was completed, the close relationship between anxiety, depression, and insomnia suggests that mental health likely plays a role in this elevation. A small but significant subset of survivors demonstrated delayed sleep onset suggestive of circadian delay; however, CNS diagnosis and cranial radiation were not significant predictors, suggesting that hypothalamic injury is not a primary mechanism of sleep concerns. Similarly, survivors exhibited an 11% increase in snoring risk. BMI was an important factor in predicting snoring, but treatment exposures were less consistent predictors, suggesting that the pulmonary mechanism of sleep concerns are only partially supported. Last, CHC burden was an important correlate of overall sleep quality, supporting the mechanism that current physical and mental health is related to sleep concerns. Pain-related sleep disturbances were also significantly more common in survivors. Further examination of these mechanisms in longitudinal samples is needed, as chronic poor sleep quality may exacerbate health conditions and pain thresholds.

Patients treated in adolescence were more likely to use sleep medication, and younger adolescents were more likely to report poor sleep quality. Although the precise etiology of sleep problems in childhood cancer survivors is not fully known and was not measured in the study, adolescents’ sleep health may be more vulnerable to cancer-related disruption, as this is a time of significant change in the sleep–wake circadian system (32). Further, the stress of cancer, anxiety, and fear of recurrence may trigger hyperarousal (33), resulting in long-lasting sleep difficulties. In individuals with an insomnia predisposition, cancer could precipitate the onset of sleep difficulties (15). Furthermore, common behavioral changes to sleep habits and patterns during childhood cancer treatment (eg, irregular/interrupted sleep schedules, relying on external sources such as technology or medication to facilitate sleep) are also known to increase the risk for poor sleep well into adulthood (34-36).

Both survivors and siblings reported using medications for sleep in the past 30 days at higher rates than a recent national survey [8.4% of adults (37) compared with 16.6% in siblings and 19.2% in survivors]. Of note, the rate of medication use in siblings is nearly double that in the general population, highlighting the potential that the impact of a childhood cancer diagnosis on a family may persist. Mounting evidence shows that sleep medications confer additional risk of physical (38) and mental health (39,40), morbidity, and overall mortality in the general population (41,42). Specifically, the risk of cancer (42) and increased rates of infection (43) are particularly relevant to adult survivors of childhood cancer. Survivorship clinicians should inquire about medications and supplements used for sleep, as those might create an interaction with medications used for chronic conditions. Nonpharmacological interventions should be offered, such as cognitive behavioral therapy for insomnia, which is the first line of treatment for chronic insomnia in adults (44), has been shown to be superior to sleep medications in a generally health population (45), and is efficacious for improving sleep in cancer survivors (46-48).

Among survivors, demographic factors were more consistent predictors of sleep than diagnosis or treatment exposures. Women and individuals with higher BMIs were at risk for poor sleep quality. Sex differences in rates of insomnia and sleep health across the lifespan have been well documented (49,50), but the exact mechanism of the increased risk remains a source of debate. Women were more likely to report insomnia symptoms and less likely to report snoring or delayed bedtimes. Our results are consistent with prior research describing significant associations of high BMI with short sleep duration, insomnia, and obstructive sleep apnea, through what is most likely a bidirectional relationship between sleep and weight (51). These demographic risk factors are consistent with research in the general population; however, the cancer experience appears to elevate this risk.

Treatment exposures were more relevant than diagnosis in predicting sleep. Specifically, the finding that neck radiation is protective against snoring is novel in pediatric cancer survivors. Almost half of patients who received neck radiation were diagnosed with Hodgkin lymphoma, followed by leukemia (20%) and CNS tumors (17%). In the multivariable model, survivors of leukemia were more likely to snore than the CNS tumor survivor reference group, whereas Hodgkin lymphoma was not a predictor of snoring. Neck radiation-induced muscle atrophy may alter the upper airway structures, increasing patency and decreasing likelihood of obstruction. The finding that high-dose radiation leads to a greater reduction in snoring risk compared with low-dose neck radiation further supports this hypothesis. Preliminary imaging data from Hodgkin lymphoma survivors also support this finding (52).

Survivors who received any abdominal radiation were at greater risk of snoring, most likely because of the impact of radiation on muscles involved in respiratory effort (53). Previous work has described an increased incidence of obstructive sleep apnea in Hodgkin lymphoma survivors who received abdominal radiation (20,54); although this diagnosis was not a predictor of snoring, 23% of the survivors who received abdominal radiation were diagnosed with Hodgkin lymphoma. Low-dose abdominal radiation was also related to a greater likelihood of poor sleep quality, snoring, and delayed bedtimes compared with those who did not receive radiation. The low-dose abdominal radiation group was made up primarily of patients with Wilm tumor (31%) or leukemia (28%), which are groups likely to be diagnosed in early childhood. Although neither age at diagnosis nor specific diagnoses were predictors of these sleep outcomes, it is possible radiation in young children confers elevated risk for disrupted sleep. The long treatment course of these types of cancer early in development when sleep skills are being solidified may impact a patient’s ability to fall asleep and remain asleep independently.

To our knowledge, this is the largest evaluation of sleep in long-term pediatric cancer survivors; however, the reliance on self-report is a limitation. Specifically, frequency of self-reported snoring may be underreported but likely reflects some known amount of snoring that has been reported by a bed partner. Prior research has demonstrated relationships between self-report of snoring and the likelihood of being diagnosed with sleep apnea (35), suggesting that such screening questions have clinical validity. Data regarding sleep medication use rely on a single question that does not differentiate between type and dose of medication (ie, hypnotic, benzodiazepine, over-the-counter sleep aid) or additional supplementation. More detailed questions regarding sleep management strategies in childhood cancer survivors should include typical and atypical sleep aids as well as supplements and cannabis to better understand frequency of use and the health consequences for cancer survivors.

Sleep concerns affect childhood cancer survivors at higher rates than sibling peers, suggesting that continued screening for symptoms of insomnia, insufficient sleep, and snoring is warranted in adult survivors of childhood cancer, especially in patients with high chronic disease burden. Childhood cancer survivors should be regularly screened for sleep concerns, and cognitive behavioral therapy for insomnia should be implemented into routine survivorship care.

## Supplementary Material

pkae010_Supplementary_Data

## Data Availability

The Childhood Cancer Survivor Study is a US National Cancer Institute funded resource (U24 CA55727) to promote and facilitate research among long-term survivors of cancer diagnosed during childhood and adolescence. CCSS data are publicly available on dbGaP at https://www.ncbi.nlm.nih.gov/gap/ through its accession number phs001327.v2.p1 and on the St Jude Survivorship Portal within the St. Jude Cloud at https://survivorship.stjude.cloud/. In addition, utilization of the CCSS data that leverages the expertise of CCSS Statistical and Survivorship research and resources will be considered on a case-by case basis. For this utilization, a research Application Of Intent followed by an Analysis Concept Proposal must be submitted for evaluation by the CCSS Publications Committee. Users interested in utilizing this resource are encouraged to visit http://ccss.stjude.org. Full analytical data sets associated with CCSS publications since January of 2023 are also available on the St. Jude Survivorship Portal at https://viz.stjude.cloud/community/cancer-survivorship-community∼4/publications.
