# Supplementary material for: Mechanisms of sleep disturbances in long-term cancer survivors: a childhood cancer survivor study report
Source: JNCI Cancer Spectr. 2024 Feb 15;8(2):pkae010. doi: 10.1093/jncics/pkae010 (PMC10932943; doi:10.1093/jncics/pkae010)
Supplement: pkae010_Supplementary_Data [file pkae010_supplementary_data.pdf]

Supplementary Material

Supplementary Figure 1. Consort Diagram of Survivors.

Supplementary Figure 2. Consort Diagram of Siblings.

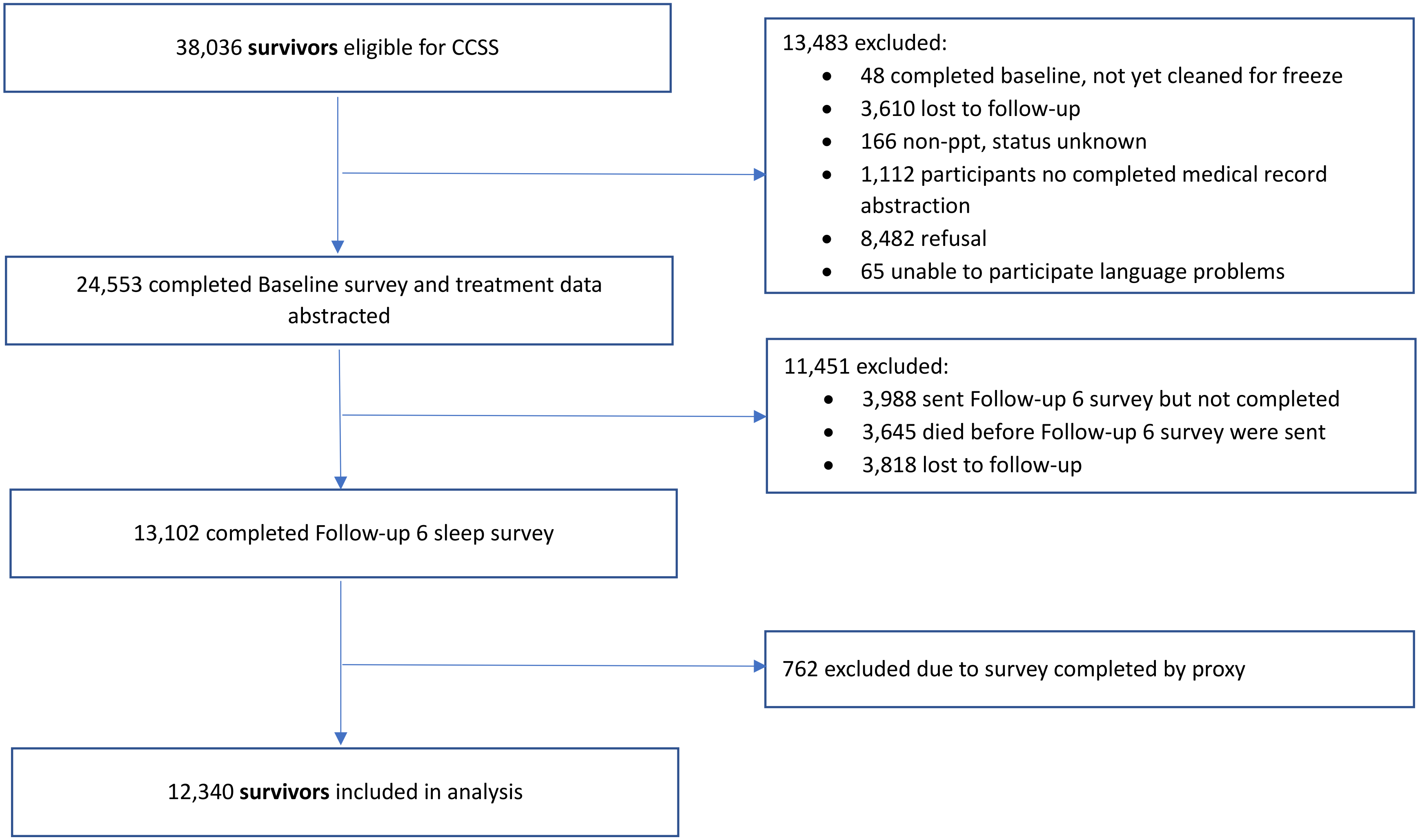
38,036 **survivors** eligible for CCSS
13,483 excluded:
48 completed baseline, not yet cleaned for freeze
3,610 lost to follow-up
166 non-ppt, status unknown
1,112 participants no completed medical record abstraction
8,482 refusal
65 unable to participate language problems
24,553 completed Baseline survey and treatment data abstracted
11,451 excluded:
3,988 sent Follow-up 6 survey but not completed
3,645 died before Follow-up 6 survey were sent
3,818 lost to follow-up
13,102 completed Follow-up 6 sleep survey
762 excluded due to survey completed by proxy
12,340 **survivors** included in analysis

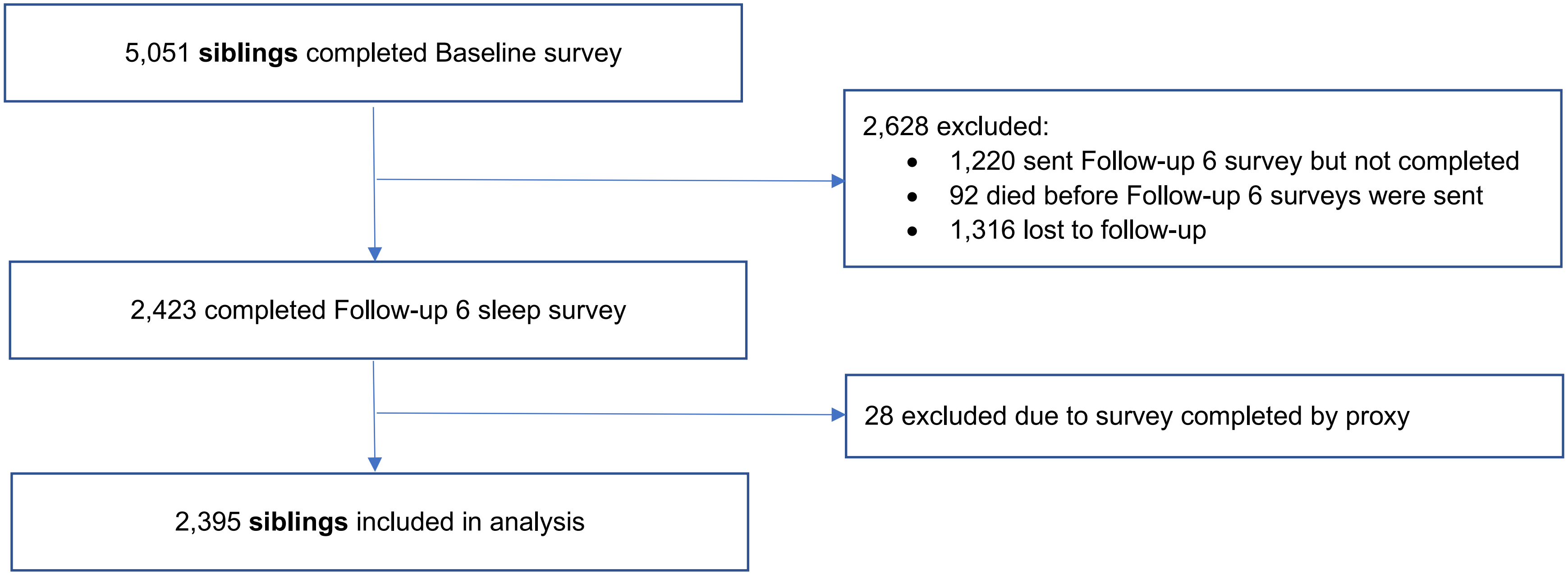
5,051 **siblings** completed Baseline survey
2,628 excluded:
- 1,220 sent Follow-up 6 survey but not completed
- 92 died before Follow-up 6 surveys were sent
- 1,316 lost to follow-up
2,423 completed Follow-up 6 sleep survey
28 excluded due to survey completed by proxy
2,395 **siblings** included in analysis

Supplementary Table.

Table 1. Multivariable models comparing frequency of sleep behaviors between survivors and siblings.

**Sleep Domain**	**Sleep Variable**	**Survivors (N=12,340) N (%)**	**Siblings (N=2,395) N (%)**	**Adjusted PR (95% CI)** [a, b, c, d]
Sleep Duration	Total sleep time <6 hours	1486 (12.0)	255 (10.6)	**1.30 (1.13 - 1.50)**
Sleep Quality	PSQI Total Score >5	5561 (45.1)	959 (40.0)	**1.20 (1.13 - 1.27)**
Insomnia Symptoms	Sleep Onset Latency >30 minutes OR 5a >3 times per week	4767 (38.8)	763 (32.0)	**1.26 (1.18 - 1.35)**
	Sleep Efficiency <85%	4021 (33.3)	699 (29.9)	**1.19 (1.10 - 1.29)**
	Night awakening/early morning awakening >3 times per week	4306 (35.6)	851 (36.0)	**1.09 (1.02 - 1.16)**
Sleep Disordered Breathing Symptoms	Snoring >3 times per week	2164 (18.0)	410 (17.4)	**1.11 (1.01 - 1.23)**
Delayed Sleep Timing	Sleep onset after 1 am	756 (6.2)	83 (3.5)	**1.78 (1.39 - 2.29)**
Sleep Medication Use	Three or more times a week	1629 (13.2)	276 (11.5)	**1.28 (1.12 - 1.45)**
Sleep disturbance due to pain	Three or more times per week	1546 (12.5)	220 (9.2)	**1.60 (1.39 - 1.84)**

a: All models use Modified Poisson model was used to directly estimate prevalence ratio (PR)

b: Siblings are the reference level

c: Adjusted for Age, sex, race and BMI. GEE used to account for within-family correction.

d: Inverse probability weighting was applied to account for the under-sampling of acute lymphoblastic leukemia survivors in the design of the CCSS expansion cohort (diagnosis in 1987-99)
